# Hyperglycemia-induced hemichorea-hemiballismus syndrome – a systematic review

**DOI:** 10.20945/2359-4292-2022-0413

**Published:** 2024-03-28

**Authors:** Mariana Costa Hoffmeister, Paola S. G. Bonavides, Vanessa Maurer Wiercinski, Viviane Alessio Baggio, Roberta de Pádua Borges, Gesner Francisco Xavier, Clara K. Maraschin, Beatriz D. Schaan

**Affiliations:** 1 Hospital de Clínicas de Porto Alegre Serviço de Endocrinologia Porto Alegre RS Brasil Serviço de Endocrinologia, Hospital de Clínicas de Porto Alegre, Porto Alegre, RS, Brasil; 2 Universidade Federal de Minas Gerais Faculdade de Medicina Belo Horizonte MG Brasil Universidade Federal de Minas Gerais, Faculdade de Medicina, Belo Horizonte, MG, Brasil; 3 Universidade Federal do Rio Grande do Sul Faculdade de Medicina Departamento de Medicina Interna Porto Alegre RS Brasil Universidade Federal do Rio Grande do Sul, Faculdade de Medicina, Departamento de Medicina Interna, Porto Alegre, RS, Brasil; 4 Universidade Federal do Rio Grande do Sul Faculdade de Medicina Departamento de Clínica Médica Porto Alegre RS Brasil Universidade Federal do Rio Grande do Sul, Faculdade de Medicina, Departamento de Clínica Médica, Programa de Pós-graduação em Ciências Médicas: Endocrinologia, Porto Alegre, RS, Brasil; 5 Instituto de Avaliação de Tecnologia em Saúde Conselho Nacional de Desenvolvimento Científico e Tecnológico Hospital de Clínicas de Porto Alegre Porto Alegre RS Brasil Instituto de Avaliação de Tecnologia em Saúde (IATS), Conselho Nacional de Desenvolvimento Científico e Tecnológico (CNPq), Hospital de Clínicas de Porto Alegre, Porto Alegre, RS, Brasil

**Keywords:** Hyperglycemia, diabetes, hemichorea-hemiballismus syndrome, basal ganglia

## Abstract

Nonketotic hyperglycemia may occur as a cause of chorea in patients with chronic decompensated diabetes. Because it is rare and consequently poorly studied, diagnosis and treatment can be delayed. Therefore, our objective was to summarize clinical and radiological features, as well as treatments performed, from previously reported cases to facilitate adequate management in clinical practice. We searched MEDLINE/PubMed, EMBASE, Cochrane, CINAHL, Web of Science, Scopus, and LILACS databases for studies published before April 23, 2021. We included case reports and case series of adults (aged ≥ 18 years) that described hyperglycemic chorea with measurement of glycated hemoglobin (HbA1c) and cranial magnetic resonance imaging (MRI). Studies were excluded if participants were pregnant women, aged < 18 years, and had no description of chorea and/or physical examination. We found 121 studies that met the inclusion criteria, for a total of 214 cases. The majority of the included studies were published in Asia (67.3%). Most patients were women (65.3%) aged > 65 years (67.3%). Almost all patients had decompensated diabetes upon arrival at the emergency department (97.2%). The most common MRI finding was abnormalities of the basal ganglia (89.2%). There was no difference in patient recovery between treatment with insulin alone and in combination with other medications. Although rare, hyperglycemic chorea is a reversible cause of this syndrome; therefore, hyperglycemia should always be considered in these cases.

## INTRODUCTION

Chorea, from the Greek word *choros*, means dance. It is a neurological disorder associated with involuntary spasmodic muscle movements. Nonketotic hyperglycemia is a rare cause of chorea. In 1960, Bedwell described the first case of severe hyperglycemia associated with hemiballismus, which resolved with correction of blood glucose (1).

Typically, hyperglycemic chorea occurs in Asian women with long-standing type 2 diabetes and chronic poor glycemic control. Cranial magnetic resonance imaging (MRI) shows a characteristic T1 hyperintensity signal in the basal ganglia (2). A recent systematic review identified 176 patients from 72 articles, of whom only 17% had newly diagnosed diabetes mellitus at first presentation (3). Another systematic review evaluated 286 patients from 136 studies and showed that 63% were women, 100% received hypoglycemic drugs, 60.84% received neuroleptics, and 84.86% showed complete resolution (4). It is not clear whether there is any standard or specific treatment for cases of chorea associated with hyperglycemia, both regarding the prescription of anticonvulsants and regarding the prescription of insulin alone or in combination with other antihyperglycemic drugs (3,5).

We report the case of a patient diagnosed with diabetes mellitus at presentation with hyperglycemia-induced hemichorea-hemiballismus syndrome. We also conducted a systematic review of clinical and radiological features, treatment, and prognosis.

## MATERIALS AND METHODS

### Information sources

We searched MEDLINE/PubMed, EMBASE, Cochrane, Cumulative Index to Nursing and Allied Health Literature (CINAHL), Web of Science, Scopus, and LILACS databases for articles published from inception to April 23, 2021. We set no language restrictions. A research librarian developed the search strategy. Our search did not include any gray literature. The complete search strategy is provided in Table 1.

**Table 1:** Search strategies

Base	Search strategy
MEDLINE/PubMed	(("Diabetes Mellitus"[Mesh] OR "Diabetes Mellitus") OR ("Diabetes Mellitus, Type 2"[Mesh] OR Ketosis-Resistant diabetes [title/abstract] OR Ketosis Resistant diabetes [title/abstract] OR Maturity-Onset diabetes [title/abstract] OR Maturity Onset diabetes [title/abstract] OR NonInsulin Dependent diabetes [title/abstract] OR Non-Insulin-Dependent diabetes [title/abstract] OR Type 2 Diabetes [title/abstract] OR stable Diabetes [title/abstract] OR Diabetes Mellitus Type II [title/abstract] OR MaturityOnset Diabetes Mellitus [title/abstract] OR Maturity Onset Diabetes Mellitus [title/abstract] OR MODY [title/abstract] OR NIDDM [title/abstract] OR Adult-Onset Diabetes Mellitus [title/abstract] OR Diabetes Mellitus Noninsulin Dependent [title/abstract] OR Hyperglycemia [title/abstract])) AND ("Chorea"[Mesh] OR "diabetic striatopathy" [title/abstract] OR "hyperglycemic hemichorea" [title/abstract] OR "hyperglycemic chorea" [title/abstract] OR Dyskinesia [title/abstract] OR hemidystonia [title/abstract] OR "involuntary movement" [title/abstract] OR "basal ganglia syndrome" [title/abstract] OR Dyskinesia [title/abstract] OR hemidystonia [title/abstract] OR "striatal hyperintensity" [title/abstract] OR "T1-weighted hyperintensity" [title/abstract])
Embase	(‘diabetes mellitus’: ti,ab,kw OR ‘non-insulin dependent diabetes mellitus’:ti,ab,kw) AND (‘chorea’/exp OR chorea OR ‘diabetic striatopathy’/exp OR ‘diabetic striatopathy’ OR ‘hyperglycemic hemichorea’ OR ‘hyperglycemic chorea’ OR ‘involuntary movement’/exp OR ‘involuntary movement’ OR ‘basal ganglia syndrome’ OR ‘dyskinesia’/exp OR dyskinesia OR ‘hemidystonia’/exp OR hemidystonia OR ‘striatal hyperintensity’ OR ‘t1-weighted hyperintensity’)
Cochrane	("Diabetes Mellitus" OR "Type 2 diabetes mellitus" OR "Non-insulin dependent diabetes mellitus") AND (Chorea OR "diabetic striatopathy" OR "hyperglycemic hemichorea" OR "hyperglycemic chorea" OR Dyskinesia OR hemidystonia OR "involuntary movement" OR "basal ganglia syndrome" OR Dyskinesia OR hemidystonia OR "striatal hyperintensity" OR "T1-weighted hyperintensity")
Web of Science	("Diabetes Mellitus" OR "Type 2 diabetes mellitus" OR "Non-insulin dependent diabetes mellitus") AND (Chorea OR "diabetic striatopathy" OR "hyperglycemic hemichorea" OR "hyperglycemic chorea" OR Dyskinesia OR hemidystonia OR "involuntary movement" OR "basal ganglia syndrome" OR Dyskinesia OR hemidystonia OR "striatal hyperintensity" OR "T1-weighted hyperintensity")
Scopus	("Diabetes Mellitus" OR "Type 2 diabetes mellitus" OR "Non-insulin dependent diabetes mellitus") AND (Chorea OR "diabetic striatopathy" OR "hyperglycemic hemichorea" OR "hyperglycemic chorea" OR Dyskinesia OR hemidystonia OR "involuntary movement" OR "basal ganglia syndrome" OR Dyskinesia OR hemidystonia OR "striatal hyperintensity" OR "T1-weighted hyperintensity")
BVS/LILACS	(TW: "Diabetes Mellitus" OR "Diabetes Mellitus Tipo 2" OR "Diabetes Mellitus, Type 2" OR "Diabetes Mellitus Tipo 2" OR "Type 2 diabetes mellitus" OR "Non-insulin dependent diabetes mellitus") AND (Chorea OR "diabetic striatopathy" OR "hyperglycemic hemichorea" OR "hyperglycemic chorea" OR Dyskinesia OR hemidystonia OR "involuntary movement" OR "basal ganglia syndrome" OR Dyskinesia OR hemidystonia OR "striatal hyperintensity" OR "T1-weighted hyperintensity")

### Study selection

The results of the database searches were compiled using Rayyan software, and two reviewers (MH and VW) independently screened titles and abstracts using a standardized form for data extraction, and then screened candidate full-text articles for selection based on our inclusion and exclusion criteria. Full texts of all potential studies for inclusion were retrieved and independently assessed by other two reviewers (PB and VB). Any disagreements between reviewers at any stage were resolved by consulting a third independent reviewer (RPB).

Studies eligible for inclusion in this review were case reports and case series of adults (aged ≥ 18 years) that described hyperglycemic chorea with measurement of glycated hemoglobin (HbA1c) and cranial MRI. Studies were excluded if participants were pregnant women, aged < 18 years, and had no description of chorea and/or physical examination. If articles were not available, we contacted the corresponding authors. Decompensated diabetes was defined as HbA1c > 8% or capillary blood glucose > 200 mg/dL.

**Figure 1:**
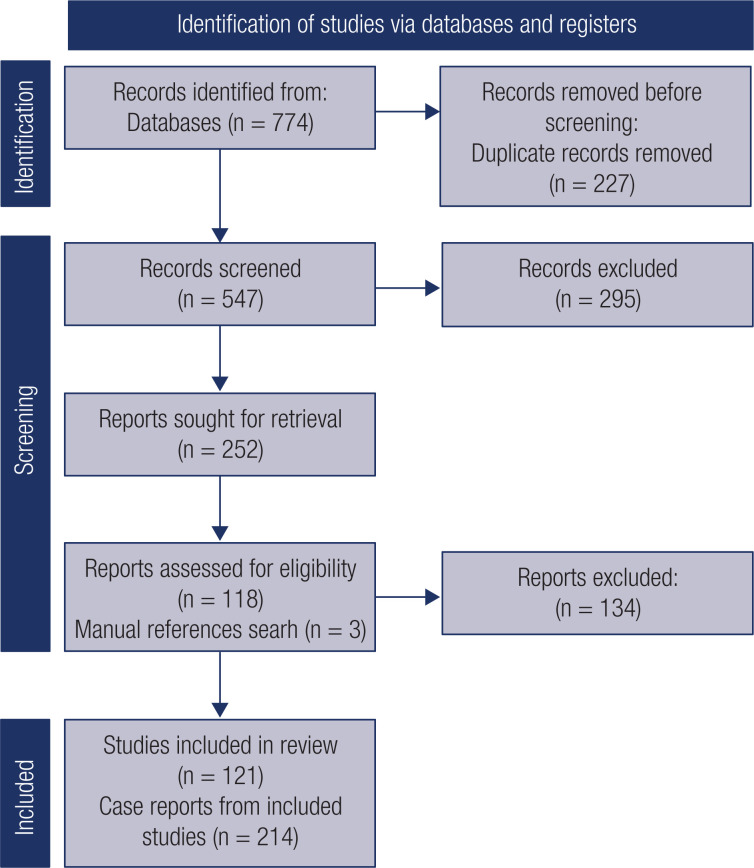
Flow diagram: Identification and selection of articles

For studies meeting eligibility, data extracted included age of patient(s), laboratory and clinical data, MRI findings, treatment performed, type of diabetes, time from diagnosis, and study country. The flowchart of studies selection process is summarized in figure 1.

### Data analysis

Narrative and quantitative syntheses were performed to describe the results. Data were analyzed in SPSS. Parametric data were presented as mean (SD), and nonparametric data as median (IQR). P values of less than 0.05 and 95% CIs were considered statistically significant.

## RESULTS

### Case report

A 62-year-old female patient was seen in the emergency department of a tertiary care hospital reporting that two days ago she started walking unsteadily and one day ago she presented uncoordinated and involuntary left hemi body movements with progressive worsening, maintaining a preserved level of consciousness. In addition, dysarthria and tremors appeared in the left hemiface. She reported a history of pre-diabetes for four years, and was taking metformin (850 mg twice a day) with no recent medical follow-up. She also presented systemic arterial hypertension and atrial fibrillation, both under treatment. Upon arrival, she was alert, oriented, sweating, capillary blood glucose was 136 mg/dL, heart rate (HR) was 140-160 bpm, normal cardiac and pulmonary auscultations, no abnormality in the abdominal exam. Intravenous diazepam 5 mg was administered with improvement. Initial laboratory tests revealed glycemia 127 mg/dL, creatinine 1.11 mg/dL (glomerular filtration rate 53 mL/min/1.73 m2), bicarbonate 16 mEq/L (reference value: 23-31 mEq/L), sodium 146 mEq/L (reference value:136-145 mEq/L), potassium 4.3 mEq/L (reference value: 3.5-5.1 mEq/L), calcium 10 mg/dL (reference value: 8.4-10.2 mg/dL), hemoglobin 12.4. Serologies for HIV, hepatitis C, hepatitis B and syphilis were all negative. Brain CT showed areas of increased attenuation in the striated bodies, suggesting the possibility of non-ketotic hyperglycemia as the cause of the symptoms, with no visible expansive lesion or intracranial collections. A lumbar puncture was performed with cerebrospinal fluid showing 1 leukocyte/uL (reference value: up to 5/uL), no germs, protein 29.6 mg/dL (reference value: up to 40 mg/dL). Electrocardiogram showed atrial fibrillation. The patient underwent brain MRI with intravenous injection of gadolinium contrast, demonstrating T1 hypersignal with a component of hypo signal in T2/FLAIR. Glycated hemoglobin (HbA1c) was also evaluated, which was 12.7% (reference value: < 5.7%). During hospitalization she was started on risperidone 2 mg twice a day, with progressive improvement of motor symptoms, later reduced to 1 mg twice a day. Metformin was also adjusted to 1,000 mg twice a day and NPH insulin was introduced twice a day, 14 IU before breakfast and 10 IU at 10 pm. Patient returned to the outpatient clinic two months after hospital discharge, reporting no symptoms, with no recurrence of hemichorea. She maintained good adherence to the treatment, presenting HbA1c 8.2%.

### Literature review

The initial search yielded 774 records, 547 of which remained after adjusting for duplicates. After title and abstract screening, 249 studies were retrieved for full-text review, 118 of which met the inclusion criteria. Another 3 full-text articles cited in the initially screened texts were considered eligible, bringing the total number of included studies to 121, for a total of 214 cases.

Table 2 shows the characteristics of patients in the reported cases, according to sex. Most patients were women (65.3%). The median age was 71 years, and 67.3% were over 65 years of age. The majority of the included studies were published in Asia (67.3%), with South Korea being the country with the largest number of reported cases (n = 45). Almost all patients had decompensated diabetes (97.2%) on hospital arrival, and in 22% of them the diagnosis was made when the patient presented to the emergency department with chorea. The most common MRI finding was abnormalities of the basal ganglia (89.2%), with only 5.2% of cases with normal MRI.

**Table 2:** Characteristics of the participants of the included studies according to sex (n = 214)

		Total N = 214	Men N = 74 (34,7)	Women N = 139 (65,3)	p
Age (years)					0.357
	≤65	69 (32.7)	27 (37)	42 (30.4)	
	>65	142 (67.3)	46 (63)	96 (69.4)	
Glucose (mg/dL)		379 (269.5-558.3)	355 (249-563)	400 (287-544)	0.430
HbA1c (%)		13.7 (10.9-15.1)	14.1 (12.5-15.6)	13.2 (10.4-14.9)	**0.014**
Uncontrolled diabetes*		206 (97.2)	73 (100)	133 (95.7)	0.096
Site of MRI change					0.689
	Basal Ganglia	190 (89.2)	65 (87.8)	125 (89.9)	
	Other	12 (5.6)	4 (5.4)	8 (5.8)	
	Normal	11 (5.2)	5 (6.8)	6 (4.3)	
Previous diabetes		153 (78.1)	50 (72.5)	103 (81.1)	0.206
Previous use of insulin		22 (27.2)	6 (20)	16 (31.4)	0.311
Continent (n)					
	Americas	41	20	21	
	Asia	144	45	99	
	Europe	23	7	16	
	Oceania	3	0	3	
Diabetes type					0.683
	1	6 (3.7)	3 (5.8)	3 (2.7)	
	2	79 (44.8)	24 (46.2)	49 (44.1)	
	Not specified	83 (50.9)	25 (48.1)	58 (52.3)	

HbA1c: glycated hemoglobin; MRI: magnetic resonance imaging. Continuous variables are expressed as mean ± standard deviation or median [interquartile range (p25-p75)]. Categorical variables are expressed as n (%). Comparisons were performed using Fisher's exact test or Mann-Whitney test.

* Uncontrolled diabetes was defined as fasting blood glucose > 200 mg/dL or HbA1c > 8%.

Regarding publication dates, there was an increase in reports in recent years: 5 case reports were published from 1994 to 2000, 25 from 2001 to 2010, 90 from 2011 to 2020, and 2 in 2021, in addition to the present case report.

Regarding patient recovery, there was no difference between treatment with insulin alone and in combination with other medications (p 1.0). Also, no difference was found between insulin alone and insulin with haloperidol, insulin with haloperidol and benzodiazepine, or insulin with benzodiazepine (p 0.358).

## DISCUSSION

Nonketotic hyperglycemic hemichorea is a poorly recognized entity, with few studies and a probably underestimated incidence. In the last two decades, the prevalence of adults with diabetes has increased from 4.6% to 10.5% of the global population (6). Likewise, there has been an increase in reports of hyperglycemic chorea published over the years. However, whether the number of diagnoses has increased or cases are just being increasingly recognized and reported remains unclear.

In our study, 61 patients (22%) presented with symptoms of chorea as the first presentation of diabetes. This is interesting as it indicates that hyperglycemia should always be suspected in these cases. A recent study showed similar data: 17% of chorea cases also had newly diagnosed diabetes (3).

It is known that long-term complications of diabetes, both microvascular and macrovascular, are more common in people with long-standing poor glycemic control (7). Although chorea cannot be considered a complication of diabetes, we observed that, regarding glycemic control, 97% of the cases in which the two conditions were associated had blood glucose levels > 200 mg/dL or HbA1c > 8% (5,8). Although the exact mechanism of chorea associated with hyperglycemia and/or diabetes is unknown, in hyperglycemia, the brain metabolism is known to shift to anaerobic pathways, with inactivation of Krebs cycle. In this setting, new substrates are used by the brain, such as gamma-aminobutyric acid (GABA), which may be involved in the genesis of basal ganglia dysfunction and result in disinhibition of the subthalamus and basal ganglia, thus leading to the involuntary movements characteristic of chorea (9). However, this hypothesis does not consider circumstances where hyperglycemia is corrected but clinical symptoms are not quickly reversed or even worsen. This may occur by a mechanism similar to that of diabetic retinopathy that worsens with rapid normalization of blood glucose levels (10).

Most reports included in this review describe patients from Asian countries, consistent with the literature (5,11,12). However, such data may be due only to reporting bias. There are no clear data in the literature with appropriate genetic analyses linking the occurrence of chorea associated with hyperglycemia to genetic or family profiles.

Diabetes is estimated to affect more than 500 million adults aged 20-79 years worldwide (13), affecting non-Hispanic whites in 7.1% of cases, Asian Americans in 8.4%, Hispanic Americans in 11.8%, non-Hispanic blacks in 12.6%, and Native Americans in 33% (14). The prevalence of diabetes is similar in men and women, being higher in those aged 75-79 years (13). Regarding complications, women with diabetes, especially postmenopausal women, tend to be at higher cardiovascular risk than men with diabetes in the same age group (15). In line with this, our study showed a higher prevalence of chorea in older women, data similar to those reported in the literature (3,5).

Population aging leads to a larger number of neurodegenerative disorders due to the accumulation of nuclear DNA (nDNA) damage to neurons in the cerebral cortex and hippocampus, as well as oxidative damage to biomolecules that lead to a chronic inflammatory response (16). Considering all these factors, we can infer that older patients with diabetes are even more likely to develop diabetes-related neurological complications, including hyperglycemic chorea.

In conclusion, recognizing this rare complication of diabetes is important, as early diagnosis and management will result in resolution of symptoms and better outcomes, avoiding unnecessary investigations.

## SUPPLEMENTARY MATERIAL

**Table:**

AUTHOR		Year	Gender	Age	Country	Glucose	HbA1c	Clinical presentation	Site of change on MRI	Chorea treatment	Recovery	Recurrence	Diabetes Diagnosis	Diabetes Type	Diabetes Duration
Abdelghany, M		2014	M	34	USA	230	13,6	Hemichorea	Basal ganglia	Ins + other	Y	N	Y	T2DM	N.I.
Abe, Y															
	Case 1	2009	F	72	Japan	130	6,2	Hemichorea	Basal ganglia	ins + hal	Y	N	Y	T2DM	<5 years
	Case 2	2009	M	73	Japan	151	17,2	Hemichorea	Basal ganglia	ins + hal	Y	N	Y	N.S.	N.I.
	Case 3	2009	F	68	Japan	118	6,5	Hemichorea	Basal ganglia	ins + hal	Y	N	N	-	-
	Case 4	2009	M	56	Japan	161	8	Other	Basal ganglia	Insulin	N.I.	N.I.	Y	N.S.	N.I.
	Case 5	2009	F	84	Japan	107	7,4	Hemichorea	Basal ganglia	ins + hal	Y	N	Y	N.S.	5-10 years
Abou-Al-Shaar, H		2018	M	59	USA	351	8,4	Other	Basal ganglia	Ins + Other	Y	N	Y	T2DM	<5 years
Acuna, MJV		2016	M	72	USA	873	13,2	Hemichorea	Normal	Insulin	Y	N	N	-	-
Ahmad, A		2013	F	63	Singapore	522	16,7	Hemichorea	Basal ganglia	Insulin	Y	N	Y	T2DM	N.I.
Ahmad, S		2018	F	83	UK	540	14,1	Hemichorea	Basal ganglia	Insulin	Y	N	Y	T2DM	N.I.
Al-Quliti, KW		2016	F	58	Saudi Arabia	540	13,5	Hemichorea	Basal ganglia	Ins + hal + bdz	Y	N	Y	N.S.	>10 years
Ari, BC		2021	F	67	Turkey	999	14	Hemichorea	Normal	ins + hal	Y	N	Y	T2DM	>10 years
Arriaga, AC															
	Case 1	2009	M	70	Mexico	202	15,4	Chorea	Basal ganglia	Ins + hal + bdz	Y	Y	N	-	-
	Case 2	2009	F	81	Mexico	375	5,6	Hemichorea	Basal ganglia	ins + hal	Y	N	N	-	-
Atay, M															
	Case 1	2014	F	79	Turkey	254	11	Hemichorea	Basal ganglia	Insulin	Y	N	Y	T2DM	N.I.
	Case 2	2014	F	75	Turkey	564	14,4	Hemichorea	Basal ganglia	Insulin	Y	N	Y	N.S.	N.I.
Awasthi, D		2012	F	21	USA	535	18,4	Hemichorea	Basal ganglia	Insulin	Y	N	N	-	-
Battist,i C															
	Case 1	2009	F	80	Italy	500	8	Hemichorea	Basal ganglia	ins + hal	P	-	N	-	-
	Case 2	2009	F	78	Italy	463	10	Hemichorea	Basal ganglia	ins + hal	Y	N	N	-	-
Bendi, VS		2018	M	68	USA	1160	13,8	Chorea	Basal ganglia	ins + bdz	P	-	Y	T2DM	N.I.
Bhagwat, NM		2013	F	71	India	650	13,5	Hemichorea	Normal	Ins + Other	Y	N	Y	T2DM	5-10 years
Billich, C		2015	M	75	Germany	320	8,3	Hemichorea	Basal ganglia	Ins + Other	Y	N	Y	T2DM	<5 years
Bizet, J		2014	F	66	USA	984	12,2	Hemichorea	Basal ganglia	ins + hal	Y	N	Y	T2DM	N.I.
Buysschaert, M		2011	F	81	Belgium	609	15,3	Hemichorea	Basal ganglia	Insulin	Y	N	N	-	-
Carrion, DM		2013	M	52	Ecuador	451	10,8	Hemichorea	Basal ganglia	Insulin	Y	Y	N	-	-
Castro, DM		2009	M	73	Argentina	107	8,5	Hemichorea	Basal ganglia	ins + bdz	Y	N	Y	T2DM	<5 years
Chalia, M		2019	M	60	USA	159	14,1	Hemichorea	Basal ganglia	Ins + Other	Y	N	Y	T2DM	>10 years
Chang, CV		2007	M	66	Brazil	588	13,9	Hemichorea	Basal ganglia	ins + hal	Y	N	N	-	-
Chang, X		2017	F	84	China	669	14	Chorea	Basal ganglia	Ins + Other	Y	N	Y	T2DM	>10 years
Cheema, H		2011	F	91	Australia	756	16,1	Hemichorea	Basal ganglia	Insulin	P	-	Y	T2DM	N.I.
Chen, H		2017	M	69	Taiwan	533	10,4	Other	Normal	Insulin	Y	N	Y	T2DM	N.I.
Cherian, A		2009	F	50	EUA	421	17,7	Hemichorea	Basal ganglia	Ins + hal + bdz	Y	N	N	-	-
Cho, HS		2018	M	70	Taiwan	415	19	Hemichorea	Normal	ins + hal	Y	N	N.I.	N.I.	N.I.
Chu, K															
	Case 1	2002	F	69	Korea	348	9,5	Hemichorea	Basal ganglia	Ins + other	Y	N	Y	N.S.	5-10 years
	Case 2	2002	F	62	Korea	370	9,7	Hemichorea	Basal ganglia	ins + hal	Y	N	Y	N.S.	<5 years
Chung, SJ		2005	F	78	Korea	473	18	Hemichorea	Basal ganglia	ins + bdz	Y	N	Y	T2DM	5-10 years
D'Angelo, R		2013	M	41	Italy	174	14	Chorea	Basal ganglia	Ins + hal + bdz	Y	N	N	-	-
Das, L															
	Case 1	2016	M	18	India	553	16,2	Hemichorea	Basal ganglia	Ins + other	Y	N	Y	T1DM	5-10 years
	Case 2	2016	M	21	India	645	15,8	Hemichorea	Basal ganglia	ins + hal	Y	N	Y	T1DM	<5 years
Fatima,M		2020	M	30	N.I.	453	15,13	Hemichorea	Basal ganglia	ins + hal	Y	N	N	-	-
Fei Xiao		2019	F	62	China	320	13,6	Hemichorea	Basal ganglia	Ins + hal + bdz	P	-	Y	T2DM	5-10 years
Felicio, AC															
	Case 1	2014	M	70	Brazil	560	6,6	Hemichorea	Basal ganglia	ins + hal	Y	N	N	-	-
	Case 2	2014	M	66	Brazil	588	13,9	Hemichorea	Basal ganglia	ins + hal	Y	N	N	-	-
Fong, SL		2019	F	76	Malaysia	558,6	10,5	Hemichorea	Basal ganglia	Ins + hal + bdz	Y	N	Y	N.S.	>10 years
Gambito, MR		2016	M	77	USA	845	16	Hemichorea	Other	Ins + other	Y	N	Y	T2DM	N.I.
González, TGP															
	Case 1	2017	F	64	Spain	417	16,5	Hemichorea	Basal ganglia	ins + bdz	Y	N	Y	N.S.	N.I.
	Case 2	2017	F	68	Spain	392	13,8	Hemichorea	Basal ganglia	Ins + hal + bdz	Y	N	N	-	-
	Case 3	2017	F	85	Spain	939	15,7	Chorea	Basal ganglia	Ins + hal + bdz	Y	N	Y	N.S.	N.I.
	Case 4	2017	M	88	Spain	196	16,1	Hemichorea	Basal ganglia	Ins + hal + bdz	Y	N	N	-	-
Guo, Y															
	Case 1	2014	F	81	China	214	9,3	Hemichorea	Normal	insulin	N.I.	N.I.	N	-	-
	Case 2	2014	F	74	China	180	10,7	Hemichorea	Basal ganglia	ins + hal	N.I.	N.I.	Y	N.S.	>10 years
	Case 3	2014	F	82	China	239	9,5	Hemichorea	Basal ganglia	ins + hal	N.I.	N.I.	N	-	-
	Case 4	2014	F	82	China	504	15,7	Hemichorea	Basal ganglia	Ins + hal + bdz	N.I.	N.I.	Y	N.S.	5-10 years
	Case 5	2014	F	65	China	380	13,7	Hemichorea	Basal ganglia	Insulin	N.I.	N.I.	Y	N.S.	5-10 years
	Case 6	2014	M	70	China	355	13,6	Hemichorea	Basal ganglia	ins + hal	N.I.	N.I.	Y	N.S.	5-10 years
	Case 7	2014	M	74	China	460	12,4	Hemichorea	Basal ganglia	ins + hal	N.I.	N.I.	Y	N.S.	<5 years
Hashimoto, K		2012	F	40	USA	791	9,5	Chorea	Basal ganglia	Insulin	P	-	Y	DM1	>10 years
Hashimoto, T															
	Case 1	1998	M	77	Japan	264	18,7	Hemichorea	Basal ganglia	ins + hal	P	-	Y	N.S.	5-10 years
	Case 2	2012	F	77	Japan	632	19	Hemichorea	Basal ganglia	ins + hal	S	N	Y	N.S.	N.I.
	Case 3	2012	F	78	Japan	455	14,4	Hemichorea	Basal ganglia	ins + hal	P	-	Y	N.S.	N.I.
Helmy, A		2019	F	71	France	417	12,4	Hemichorea	Basal ganglia	Ins + Other	P	-	Y	T2DM	N.I.
Hiesgen, J		2014	M	63	Germany	258	14,4	Hemichorea	Basal ganglia	Ins + other	Y	N	Y	T2DM	N.I.
Higa, M		2003	F	82	Japan	306	13,2	Hemichorea	Basal ganglia	Insulin	Y	N.I.	Y	T2DM	5-10 years
Homaida, M		2021	F	71	UK	360	11,7	Hemichorea	Basal ganglia	ins + hal	Y	N	Y	T2DM	N.I.
Hsiao PJ		2019	F	39	Taiwan	765	13,4	Hemichorea	Basal ganglia	ins + bdz	Y	N	Y	T2DM	N.I.
Hussaini, S		2019	M	50	Netherlands	999	17,7	Other	Basal ganglia	N.I.	Y	N	Y	T2DM	N.I.
Kammeyer, RM;		2020	M	61	USA	414	14	Hemichorea	Basal ganglia + Other	Ins + haldol + other	P	-	Y	T2DM	>10 years
Kandiah N		2009	M	75	Singapore	234	14,1	Hemichorea	Basal ganglia	N.I.	N.I.	N.I.	Y	N.S.	N.I.
	Case 1	2009	M	80	Singapore	21	12,4	Hemichorea	Basal ganglia	N.I.	N.I.	N.I.	N	-	-
	Case 2	2009	F	90	Singapore	378	13	Hemichorea	Basal ganglia	N.I.	N.I.	N.I.	N	-	-
	Case 3	2009	F	78	Singapore	558	14,2	Hemichorea	Basal ganglia	N.I.	N.I.	N.I.	Y	N.S.	N.I.
	Case 4	2009	M	81	Singapore	504	16,3	Hemichorea	Basal ganglia	N.I.	N.I.	N.I.	N	-	-
	Case 5	2009	M	73	Singapore	234	10,9	Hemichorea	Basal ganglia	N.I.	N.I.	N.I.	Y	N.S.	N.I.
Kang, JH		2005	F	28	Korea	481	12,2	Hemichorea	Basal ganglia	insulin	P	-	Y	N.S.	5-10 years
Karau, P		2020	F	52	Netherlands	594	11	Hemichorea	Basal ganglia	ins + hal	Y	N	N	-	-
Kashiura, M		2017	F	87	Australia	1125	8,5	Hemichorea	Basal ganglia	ins + hal	Y	N	Y	N.S.	N.I.
Kitagawa, M		2017	M	85	Japan	563	17	Other	Basal ganglia	Ins + other	P	-	Y	N.S.	N.I.
Koh, Yh		2007	M	76	Singapore	617,4	15	Hemichorea	Basal ganglia	Insulin	Y	N	Y	T1DM	N.I.
Kranick, SM		2008	F	52	USA	575	17,7	Hemichorea	Basal ganglia	Insulin	Y	N	Y	T1DM	N.I.
Kumar Vadi, S		2020	F	70	India	268	14,5	Hemichorea	Basal ganglia	N.I.	P	-	Y	T2DM	>10 years
Labbad, I		2020	F	53	Syria	441	12,9	Hemichorea	Basal ganglia	Insulin	Y	N	N	-	-
Lancellotti, G		2015	M	86	France	306	11,9	Hemichorea	Basal ganglia + other	Insulin	Y	Y	Y	N.S.	N.I.
Lee, BC															
	Case 1	1999	F	78	Korea	216	11,3	Hemichorea	Basal ganglia	ins + hal	Y	N.I.	Y	N.S.	<5 years
	Case 2	1999	F	77	Korea	232	11,2	Hemichorea	Basal ganglia	Ins + hal + bdz	Y	N.I.	Y	N.S.	<5 years
	Case 3	1999	F	54	Korea	73	8,5	Hemichorea	Basal ganglia	Ins + hal	Y	N.I.	Y	N.S.	5-10 years
	Case 4	1999	F	77	Korea	387	15,3	Hemichorea	Basal ganglia	Ins + hal + bdz	Y	N.I.	N	-	-
	Case 5	1999	F	80	Korea	500	10	Hemichorea	Basal ganglia	Insulin	Y	N.I.	N	-	-
	Case 6	1999	F	70	Korea	359	15	Hemichorea	Basal ganglia	ins + hal	Y	N.I.	Y	N.S.	>10 years
	Case 7	1999	F	56	Korea	488	19,2	Hemichorea	Basal ganglia	insulin	Y	N.I.	Y	N.S.	>10 years
Lee, D															
	Case 1	2016	F	66	Korea	397	10	Hemichorea	Basal ganglia	Ins + hal + bdz	N.I.	N.I.	N.I.	N.I.	N.I.
	Case 2	2016	F	67	Korea	587	15	Hemichorea	Basal ganglia	insulin	Y	N.I.	N.I.	N.I.	N.I.
	Case 3	2016	F	69	Korea	167	5,9	Hemichorea	Basal ganglia	ins + bdz	N.I.	N.I.	N.I.	N.I.	N.I.
	Case 4	2016	F	71	Korea	124	7	Hemichorea	Basal ganglia	ins + bdz	Y	N.I.	N.I.	N.I.	N.I.
	Case 5	2016	F	74	Korea	532	14	Hemichorea	Basal ganglia	ins + hal	N.I.	N.I.	N.I.	N.I.	N.I.
	Case 6	2016	F	75	Korea	152	7,4	Hemichorea	Basal ganglia	ins + bdz	Y	N.I.	N.I.	N.I.	N.I.
	Case 7	2016	F	75	Korea	267	14,9	Hemichorea	Basal ganglia	Ins + hal + bdz	N.I.	N.I.	N.I.	N.I.	N.I.
	Case 8	2016	F	80	Korea	371	11,3	Hemichorea	Basal ganglia	insulin	Y	N.I.	N.I.	N.I.	N.I.
	Case 9	2016	M	80	Korea	239	14	Hemichorea	Basal ganglia	ins + bdz	Y	N.I.	N.I.	N.I.	N.I.
	Case 10	2016	F	81	Korea	569	10	Hemichorea	Basal ganglia	ins + bdz	Y	N.I.	N.I.	N.I.	N.I.
	Case 11	2016	F	81	Korea	205	13	Hemichorea	Basal ganglia	ins + bdz	Y	N.I.	N.I.	N.I.	N.I.
Lee, D (2)		2016	F	999	Korea	999	16,2	Hemichorea	Basal ganglia	insulin	Y	Y	N.I.	N.S.	N.I.
Lee, EJ															
	Case 1	2002	F	74	USA	264	9,7	Hemichorea	Basal ganglia	Ins + hal + bdz	Y	N.I.	Y	N.S.	N.I.
	Case 2	2002	F	47	USA	280	14,4	Hemichorea	Basal ganglia	Ins + hal + bdz	Y	N.I.	Y	N.S.	N.I.
	Case 3	2002	F	43	USA	315	6,1	Hemichorea	Basal ganglia	N.I.	Y	N.I.	Y	N.S.	N.I.
	Case 4	2002	F	81	USA	170	9,3	Hemichorea	Basal ganglia	ins + hal	Y	N.I.	Y	N.S.	N.I.
	Case 5	2002	F	62	USA	283	13,2	Hemichorea	Basal ganglia	ins + hal	Y	N.I.	Y	N.S.	N.I.
	Case 6	2002	F	57	USA	305	11,4	Hemichorea	Basal ganglia	Ins + hal + bdz	Y	N.I.	Y	N.S.	N.I.
Lee, P															
	Case 1	2015	F	58	Singapore	1003	16,3	Hemichorea	Other	Insulin	Y	N	Y	T2DM	>10 years
	Case 2	2015	F	76	Singapore	412	12,3	Hemichorea	Basal ganglia	Insulin	N.I.	N.I.	Y	T2DM	N.I.
Lee, SH												N.I.			
	Case 1	2011	F	60	Korea	738	13,5	Hemichorea	Basal ganglia	Insulin	Y	N.I.	Y	N.S.	5-10 years
	Case 2	2011	F	71	Korea	417	11,9	Hemichorea	Basal ganglia	Ins + hal + bdz	Y	N.I.	Y	N.S.	>10 years
	Case 3	2011	M	90	Korea	325	16,6	Hemichorea	Basal ganglia	Ins + hal + bdz	Y	N.I.	Y	N.S.	<5 years
	Case 4	2011	F	80	Korea	456	13,7	Hemichorea	Other	Ins + hal + bdz	Y	N.I.	Y	N.S.	>10 years
	Case 5	2011	F	65	Korea	367	15,4	Hemichorea	Basal ganglia	insulin	Y	N.I.	Y	N.S.	<5 years
	Case 6	2011	F	76	Korea	428	15,6	Hemichorea	Basal ganglia	Ins + hal + bdz	Y	N.I.	Y	N.S.	>10 years
	Case 7	2011	F	62	Korea	269	14,5	Hemichorea	Basal ganglia	Ins + hal + bdz	Y	N.I.	Y	N.S.	>10 years
	Case 8	2011	F	86	Korea	307	14,8	Hemichorea	Basal ganglia	ins + hal	Y	N.I.	Y	N.S.	>10 years
	Case 9	2011	F	76	Korea	176	12,3	Hemichorea	Other	ins + hal	Y	N.I.	Y	N.S.	<5 years
	Case 10	2011	F	56	Korea	402	12,2	Chorea	Other	Insulin	Y	N.I.	Y	N.S.	<5 years
	Case 11	2011	F	82	Korea	208	12,4	Hemichorea	Other	ins + hal	Y	N.I.	Y	N.S.	>10 years
	Case 12	2011	F	80	Korea	579	13,8	Hemichorea	Basal ganglia	ins + hal	Y	N.I.	Y	N.S.	> 10 years
	Case 13	2011	M	73	Korea	249	11,2	Hemichorea	Other	ins + hal	Y	N.I.	Y	N.S.	>10 years
	Case 14	2011	F	88	Korea	373	14,1	Hemichorea	Other	ins + bdz	Y	N.I.	Y	N.S.	5-10 years
	Case 15	2011	M	80	Korea	968	14	Hemichorea	Basal ganglia	Ins + Other	N.I.	N.I.	Y	N.S.	5-10 years
	Case 16	2011	M	78	Korea	640	14,8	Hemichorea	Basal ganglia	ins + bdz	Y	N.I.	Y	N.S.	<5 years
	Case 17	2011	F	84	Korea	537	10,4	Hemichorea	Other	ins + bdz	Y	N.I.	Y	N.S.	<5 years
	Case 18	2011	M	68	Korea	939	15,9	Hemichorea	Other	Insulin	Y	N.I.	N	-	-
	Case 19	2011	M	74	Korea	214	14,5	Hemichorea	Basal ganglia	ins + hal	Y	N.I.	Y	N.S.	>10 years
	Case 20	2011	M	57	Korea	790	15,1	Hemichorea	Other	Insulin	Y	N.I.	N	-	-
Lin, CJ		2017	M	73	Taiwan	200	17,3	Hemichorea	Basal ganglia	Ins + hal + bdz	Y	N.I.	Y	N.I.	N.I.
Lin, JB		2019	F	20	Singapore	999	14	Hemichorea	Basal ganglia	ins + bdz	Y	N	Y	T1DM	>10 years
Lucassen, EB		2017	F	57	USA	999	14	Hemichorea	Basal ganglia	Ins + Other	P	N	Y	T2DM	N.I.
Madu, E		2015	F	67	USA	392	7,5	Hemichorea	Basal ganglia	ins + bdz	P	N.I.	Y	T2DM	N.I.
Malzberg, GW		2020	M	79	USA	742	14,3	Chorea	Basal ganglia	Insulin	Y	N.I.	Y	T2DM	N.I.
Marmolejo, JPG															
	Case 1	2020	M	64	Colombia	377	13,8	Hemichorea	Basal ganglia	ins + hal	N	N.I.	Y	T2DM	<5 years
	Case 2	2020	F	66	Colombia	410	14	Hemichorea	Basal ganglia	ins + hal	Y	N.I.	Y	T2DM	N.I.
Massaro, F		2012	F	82	Italy	220	14,5	Chorea	Basal ganglia	Ins + other	Y	N	N	-	-
Matsuda, M		2001	F	76	Japan	264	8,2	Hemichorea	Basal ganglia	insulin	Y	Y	Y	N.S.	>10 years
Mihaela, B.V.		2011	M	62	France	432	12,6	Hemichorea	Basal ganglia	Ins + Other	Y	N	N	-	-
Modica, M.D		2015	F	71	USA	1013	16,3	Hemichorea	Normal	Insulin	Y	N.I.	Y	T2DM	N.I.
Mushtaq, U.		2016	F	71	USA	600	16	Chorea	Normal	Insulin	Y	N	Y	T2DM	<5 years
Nabatame, H		1994	F	78	Japan	401	15,1	Hemichorea	Basal ganglia	ins + hal	Y	N.I.	Y	N.S.	N.I.
Nakano, N		2005	M	65	Japan	96	10,9	Hemichorea	Basal ganglia	Ins + hal + bdz	Y	N.I.	Y	N.S.	<5 years
Nath, J		2006	F	50	USA	421	17,7	Hemichorea	Basal ganglia	Ins + hal + bdz	Y	N	N	-	-
Neupane, P.		2020	F	50	USA	1147	14	Hemichorea	Normal	Insulin	Y	N	N	-	-
Nishio, S		2015	M	68	Japan	596	14,4	Hemichorea	Basal ganglia	ins + hal	Y	N	Y	T2DM	<5 years
Ogawa, K		2008	F	73	Japan	611	11,7	Hemichorea	Basal ganglia	Ins + other	Y	N.I.	Y	N.S.	5-10 years
Ohara, S		2001	M	92	Japan	320	17,4	Hemichorea	Basal ganglia	ins + hal	N	-	Y	N.S.	N.I.
Ohmori, H															
	Case 1	2005	F	85	Japan	324	14,8	Hemichorea	Basal ganglia	Ins + other	Y	N.I.	Y	N.S.	>10 years
	Case 2	2005	F	52	Japan	659	16,3	Hemichorea	Basal ganglia	ins + hal	N	-	Y	N.S.	>10 years
Özdilek, B		2012	F	58	Turkey	495	11	Chorea	Basal ganglia	ins + hal	P	-	N	-	-
Ozgür, A		2014	M	55	Turkey	246	15,4	Hemichorea	Basal ganglia	Insulin	N.I.	N.I.	Y	N.S.	N.I.
Padmanabhan, S		2013	F	76	Australia	439	17,3	Hemichorea	Basal ganglia	Insulin	Y	N	N	-	-
Panginikkod, S		2018	M	74	USA	450	13,6	Hemichorea	Basal ganglia	ins + bdz	Y	N	Y	T2DM	>10 years
Priola, AM		2014	F	87	Italy	410	18	Hemichorea	Basal ganglia	Insulin	Y	N	Y	T2DM	N.I.
Qiang, W		2020	M	71	China	756	14	Hemichorea	Normal	Insulin	Y	N	N	-	-
Quach, T.		2020	M	64	USA	254	14,6	Hemichorea	Basal ganglia	ins + bdz	P	-	Y	T2DM	N.I.
Raza, HK		2017	F	67	China	242	13,2	Chorea	Basal ganglia	Ins + other	Y	N.I.	N	-	-
Rodrigues, RK		2019	F	68	Brazil	330	9,9	Hemichorea	Basal ganglia	Ins + hal + bdz	P	-	Y	T2DM	N.I.
Roy, U		2016	M	52	India	354	16,2	Hemichorea	Basal ganglia	ins + hal	N	-	N	-	-
Sahu, D		2018	M	64	India	490	14,4	Hemichorea	Basal ganglia	ins + hal	Y	N.I.	N	-	-
Saleh, MM		2002	M	54	USA	256	10,5	Chorea	Normal	ins + hal	P	-	N	-	-
Satish, PV		2017	F	81	India	400	17,4	Hemichorea	Basal ganglia	ins + hal	Y	N	Y	T2DM	N.I.
Selçuk,F		2016	F	49	Turkey	400	12,1	Hemichorea	Other	ins + hal	Y	Y	Y	T2DM	5-10 years
Shafran, I		2016	F	49	Israel	340	13,2	Hemichorea	Basal ganglia	N.I.	Y	N	Y	N.S.	N.I.
Shalini, B															
	Case 1	2010	M	57	Malaysia	344	12,5	Hemichorea	Basal ganglia	ins + hal	P	-	Y	N.S.	<5 years
	Case 2	2010	F	56	Malaysia	230	13,6	Hemichorea	Basal ganglia	ins + hal	P	-	Y	N.S.	>10 years
	Case 3	2010	F	76	Malaysia	304	10,5	Hemichorea	Basal ganglia	ins + hal	P	-	Y	N.S.	5-10 years
Shin, HW		2014	F	88	Korea	288	10,5	Hemichorea	Basal ganglia	ins + hal	P	-	Y	N.S.	>10 years
Slabu, H		2011	M	999	Canada	306	15,6	Hemichorea	Basal ganglia	Ins + outro	P	-	Y	T2DM	<5 years
Sohn, S.H		2011	F	67	Korea	398	10	Hemichorea	Basal ganglia	ins + hal	Y	N.I.	Y	N.S.	>10 years
Sperling, M		2018	M	63	USA	339	9,9	Hemichorea	Basal ganglia	Insulin	Y	N.I.	Y	N.S.	>10 years
Striano, P		2011	F	63	Italy	333	9	Hemichorea	Basal ganglia	ins + hal	P	-	Y	N.S.	<5 years
Su, CS															
	Case 1	2012	M	63	Taiwan	360	19	Hemichorea	Basal ganglia	Insulin	N	-	Y	T2DM	N.I.
	Case 2	2012	M	72	Taiwan	800	13,5	Hemichorea	Basal ganglia	Insulin	N	-	Y	T2DM	N.I.
	Case 3	2012	M	70	Taiwan	999	14,5	Hemichorea	Basal ganglia	ins + hal	N	-	Y	T2DM	N.I.
	Case 4	2012	M	72	Taiwan	999	7,3	Hemichorea	Basal ganglia	ins + hal	Y	N.I.	Y	T2DM	N.I.
	Case 5	2012	F	72	Taiwan	426	12,9	Hemichorea	Basal ganglia	ins + hal	N	-	Y	T2DM	N.I.
	Case 6	2012	F	66	Taiwan	400	8,3	Hemichorea	Basal ganglia	ins + hal	N	-	Y	T2DM	N.I.
	Case 7	2012	F	73	Taiwan	800	14,3	Hemichorea	Basal ganglia	ins + hal	N	-	Y	T2DM	N.I.
	Case 8	2012	F	83	Taiwan	200	8,6	Hemichorea	Basal ganglia	ins + hal	Y	N.I.	Y	T2DM	N.I.
	Case 9	2012	F	64	Taiwan	700	15,4	Hemichorea	Basal ganglia	ins + hal	Y	Y	Y	T2DM	N.I.
	Case 10	2012	F	83	Taiwan	255	13	Hemichorea	Basal ganglia	ins + hal	Y	N.I.	Y	T2DM	N.I.
	Case 11	2012	F	82	Taiwan	316	10,3	Hemichorea	Basal ganglia	ins + hal	Y	N.I.	Y	T2DM	N.I.
	Case 12	2012	F	80	Taiwan	450	10,2	Hemichorea	Basal ganglia	Insulin	Y	N.I.	Y	T2DM	N.I.
	Case 13	2012	F	65	Taiwan	300	12,5	Hemichorea	Basal ganglia	Ins + other	Y	N.I.	Y	T2DM	N.I.
	Case 14	2012	F	68	Taiwan	322	8,5	Hemichorea	Basal ganglia	ins + hal	Y	N.I.	Y	T2DM	N.I.
	Case 15	2012	F	83	Taiwan	999	13,4	Hemichorea	Basal ganglia	ins + hal	Y	N.I.	Y	T2DM	N.I.
	Case 16	2012	F	71	Taiwan	700	16,1	Hemichorea	Basal ganglia	Insulin	Y	N.I.	Y	T2DM	N.I.
	Case 17	2012	F	18	Taiwan	367	12,9	Hemichorea	Basal ganglia	ins + hal	Y	N.I.	Y	T2DM	N.I.
	Case 18	2012	M	80	Taiwan	451	13	Hemichorea	Basal ganglia	ins + hal	Y	N.I.	Y	T2DM	N.I.
	Case 19	2012	F	67	Taiwan	396	16,3	Hemichorea	Basal ganglia	Insulin	Y	N.I.	Y	T2DM	N.I.
	Case 20	2012	M	65	Taiwan	658	7,5	Hemichorea	Basal ganglia	ins + hal	Y	N.I.	Y	T2DM	N.I.
	Case 21	2012	F	50	Taiwan	345	6,1	Hemichorea	Basal ganglia	ins + hal	Y	N.I.	Y	T2DM	N.I.
Suemaru, D		2015	F	83	Japan	950	15,1	Chorea	Basal ganglia	Insulin	Y	N.I.	N	LADA	N.I.
Suzuki		2013	F	66	Japan	600	13,3	Hemichorea	Basal ganglia	Ins + Other	Y	N	Y	N.S.	>10 years
Taguchi, Y		2010	M	76	Japan	348	15,1	Hemichorea	Basal ganglia	Insulin	N.I.	N.I.	N	-	-
Teodoro, T		2014	F	68	Portugal	500	15,8	Chorea	Basal ganglia	Ins + other	P	-	Y	N.S.	N.I.
Tidehag, L		2019	F	85	Sweden	1036	14,6	Hemichorea	Basal ganglia	ins + hal	Y	N.I.	N	-	-
Tsai, MC		2014	F	79	Taiwan	309	14,4	Hemichorea	Basal ganglia	Insulin	Y	N	N	-	-
Tung, CS		2010	M	56	Taiwan	400	13,8	Other	Basal ganglia	Ins + other	P	-	Y	N.S.	5-10 years
Tyndall, EK		2020	N.I.	66	Italy	999	9,9	Hemichorea	Basal ganglia	Ins + hal + bdz	P	-	Y	N.S.	N.I.
Valenti, R		2012	F	75	Italy	270	13	Hemichorea	Basal ganglia	Ins + other	P	-	Y	T2DM	5-10 years
Vasudevan, V		2018	M	64	India	409	14,1	Hemichorea	Basal ganglia	ins + hal	P	-	N	-	-
Wang, DM		2020	F	65	China	273	16,8	Hemichorea	Basal ganglia	ins + bdz	Y	Y	Y	T2DM	>10 years
Wang, W		2020	F	79	China	600	17,85	Hemichorea	Basal ganglia	ins + hal	Y	N.I.	Y	T2DM	>10 years
Wu, MN		2014	M	74	Taiwan	312	9	Hemichorea	Basal ganglia	Ins + hal + bdz	N	-	Y	N.S.	N.I.
Yahikozawa, H															
	Case 1	1994	M	77	Japan	264	18,7	Hemichorea	Basal ganglia	ins + hal	P	-	Y	N.S.	5-10 years
	Case 2	1994	M	77	Japan	243	14,4	Hemichorea	Basal ganglia	Ins + other	P	-	Y	N.S.	>10 years
	Case 3	1994	M	92	Japan	320	17,4	Hemichorea	Basal ganglia	ins + hal	Y	N	Y	N.S.	<5 years
Yokoi, K		2017	F	77	Japan	732	12,2	Hemichorea	Basal ganglia	ins + hal	Y	N	Y	N.S.	N.I.
Zétola, VF		2010	M	75	Brazil	600	14,4	Hemichorea	Basal ganglia	Insulin	Y	Y	Y	N.S.	<5 years
Zheng, W		2020	F	58	China	219	14,5	Hemichorea	Basal ganglia	ins + hal	Y	N	Y	T2DM	>10 years
Ziemann, U		2000	F	65	Germany	209	10,4	Hemichorea	Basal ganglia	ins + hal	P	-	Y	N.S.	<5 years

MRI: magnetic resonance imaging; T1DM: type 1 diabetes mellitus; T2DM: type 2 diabetes mellitus; LADA: latent autoimmune diabetes in adults; N. I.: not informed; N.A. not applicable; ins: insulin; hal: Haldol; bdz: benzodiazepine; Y: yes; N: no; P: partial; M: male; F: female; N. S.: not specified; UK: United Kingdom; USA: United States of America.
